# 1297. Implementation of a Prosthetic Joint Infection Clinic in a Rural Setting

**DOI:** 10.1093/ofid/ofad500.1136

**Published:** 2023-11-27

**Authors:** John D Lindsay, Allison Lastinger

**Affiliations:** WVU Medicine, Morgantown, West Virginia; West Virginia University Hospital, Morgantown, West Virginia

## Abstract

**Background:**

Nearly one million patients undergo hip or knee replacements in the United States every year This number is expected to double by 2030. The rate of prosthetic joint infection (PJI) ranges from 0.5 to 2% which translates to between 50,000 to 200,000 PJIs annually. Prosthetic joint infections have high rates of morbidity and mortality. These complex patients are best managed in a multidisciplinary clinic. In 2018, we implemented a specialized Ortho ID clinic where PJI patients are seen by their joint surgeon and ID physician in a single coordinated visit. In this study, we compared no-show rate, miles traveled to clinic, C diff rates, and other complication rates for PJI patients before and after the implementation of our Ortho ID clinic.

**Methods:**

We performed a chart review of PJI patients treated at our institution, comparing a group of patients who were treated prior 2018 in the General ID clinic to a group of patients who were treated after 2018 in the Ortho ID clinic. We assessed no-show rates, cumulative distance driven and time spent driving (calculated using Google Maps), C. diff rate, and rates of side effects between the 2 groups.

**Results:**

The combination clinic improved ID follow-up. Prior to the clinic, only 70% of PJI patients were followed by ID after hospital discharge compared to 100% after the clinic was founded. The no-show rate for PJI patients decreased from 18.2% pre-clinic to 6.9% post-clinic. The mean distance traveled pre-clinic was 226 miles compared to 192 miles post-clinic. The C diff rate decreased from 9.09% pre-clinic to 5.22% post-clinic. Rate of acute kidney injury decreased from 11.1% pre-clinic to 3.45% post-clinic.

**Conclusion:**

Our Ortho ID Clinic’s biggest impact was improving ID follow-up for these complicated patients. Prior to the Clinic’s implementation, approximately 30% of PJI patients never followed up with ID after being discharged from the hospital. We saw positive outcomes with regards to reducing C diff rates and rates of acute kidney injury.

**Disclosures:**

**All Authors**: No reported disclosures

